# The two-stage interaction of Ebola virus VP40 with nucleoprotein results in a switch from viral RNA synthesis to virion assembly/budding

**DOI:** 10.1007/s13238-020-00764-0

**Published:** 2020-11-03

**Authors:** Linjuan Wu, Dongning Jin, Dan Wang, Xuping Jing, Peng Gong, Yali Qin, Mingzhou Chen

**Affiliations:** 1 State Key Laboratory of Virology and Modern Virology Research Center, College of Life Sciences, Wuhan University, Wuhan, 430072 China; 2 Wuhan Institute of Virology, Chinese Academy of Sciences, Wuhan, 430071 China

**Keywords:** Ebola virus, nucleoprotein, matrix protein, two-stage interaction, RNA synthesis, nucleocapsid, assembly/budding

## Abstract

Ebola virus (EBOV) is an enveloped negative-sense RNA virus and a member of the filovirus family. Nucleoprotein (NP) expression alone leads to the formation of inclusion bodies (IBs), which are critical for viral RNA synthesis. The matrix protein, VP40, not only plays a critical role in virus assembly/budding, but also can regulate transcription and replication of the viral genome. However, the molecular mechanism by which VP40 regulates viral RNA synthesis and virion assembly/budding is unknown. Here, we show that within IBs the N-terminus of NP recruits VP40 and is required for VLP-containing NP release. Furthermore, we find four point mutations (L692A, P697A, P698A and W699A) within the C-terminal hydrophobic core of NP result in a stronger VP40–NP interaction within IBs, sequestering VP40 within IBs, reducing VP40–VLP egress, abolishing the incorporation of NC-like structures into VP40–VLP, and inhibiting viral RNA synthesis, suggesting that the interaction of N-terminus of NP with VP40 induces a conformational change in the C-terminus of NP. Consequently, the C-terminal hydrophobic core of NP is exposed and binds VP40, thereby inhibiting RNA synthesis and initiating virion assembly/budding.

**Electronic supplementary material:**

The online version of this article (10.1007/s13238-020-00764-0) contains supplementary material, which is available to authorized users.

## Introduction

The Ebola virus (EBOV) is an enveloped non-segmented negative strand RNA virus (NNSV) and a member of the filovirus family, which consists of three genera, *Ebolavirus*, *Marburgvirus*, and *Cuevavirus*. The species *Zaire*, *Sudan*, *Bundibugyo*, *Tai Forest*, and *Reston ebolavirus* are found within the genus of *Ebolavirus*. *Marburgvirus* and *Cuevavirus* each include one species: *Marburg virus* (MARV) and *Lloviu virus* (LLOV), respectively (Negredo et al., 2011; Misasi and Sullivan, 2014; Martin et al., 2016; Yang et al., 2019). EBOV can cause a severe fever with a high fatality rate. The outbreak of EBOV in the Democratic Republic Congo from August 2018 to November 2019 caused 3,296 infections and 2,196 deaths (67%), making it the second largest outbreak since the 2014–2016 outbreak in West Africa (Hartman et al., 2010; Messaoudi et al., 2015; Vetter et al., 2016; Aruna et al., 2019; Hoenen et al., 2019). The EBOV genome is approximately 19 kb in length, encoding seven structural proteins: nucleoprotein (NP), cofactor of polymerase L (VP35), matrix protein (VP40), glycoprotein (GP), transcription activator (VP30), minor matrix protein (VP24), and RNA-dependent RNA polymerase (L) (Kirchdoerfer et al., 2017).

The viral genome of EBOV is encapsidated by NP to form an NP–RNA template that then interacts with the RNA-dependent RNA polymerase complex consisting of VP35, VP30, and L to initiate viral transcription and replication (Muhlberger et al., 1998; Weik et al., 2002; Hartlieb et al., 2007; Groseth et al., 2009; Jasenosky et al., 2010; Trunschke et al., 2013). Similar to many negative strand RNA viruses (NSVs), EBOV can form IBs, which are the viral RNA synthesis factories in infected cells (Hoenen et al., 2012; Nanbo et al., 2013). For some NNSVs, such as vesicular stomatitis virus (VSV), human respiratory syncytial virus (hRSV), and human parainfluenza virus type 3 (HPIV3), the co-expression of NP and phosphoprotein (P) is the minimum requirement for the formation of viral IBs (Garcia-Barreno et al., 1996; Heinrich et al., 2010; Zhang et al., 2013; Richard et al., 2018). By contrast, regarding EBOV, NP expression alone can lead to the formation of IBs (Nelson et al., 2016). Moreover, NP along with VP35 and VP24 are indispensable for the formation of nucleocapsid (NC)-like structures (Huang et al., 2002; Noda et al., 2007a; Shi et al., 2008; Bharat et al., 2012). In addition, a study suggests that NC is formed at the periphery of the IBs, which indicates that NP can recruit other viral proteins for viral replication and NC assembly (Nanbo et al., 2013). This suggests that NP plays a critical role in the lifecycle of EBOV. Like the NP of other NNSVs, the Ebola NP consists of a highly conserved N-terminus, which is critical for NP oligomerization and RNA binding, and a hypervariable C-terminus, which is involved in the association with VP40 and the incorporation of NCs into virions. The C-terminus of NP has no homology with any known proteins, which suggests that the NP C-terminus plays a critical role in protein–protein interactions (Buchholz et al., 1993; Longhi et al., 2003; Watanabe et al., 2006; Noda et al., 2007b; Noda et al., 2010; Dziubanska et al., 2014; Zhang et al., 2017).

Assembly and budding are key steps in the viral lifecycle. For many enveloped viruses, the virus-like particle (VLP) system is useful for studying the mechanisms of viral assembly and budding. The matrix (M) protein is indispensable for the formation and release of VLPs. For most NNSVs, like HPIV1 (Coronel et al., 1999), Sendai virus (SeV) (Sakaguchi et al., 1999; Sugahara et al., 2004), Measles virus (MeV) (Runkler et al., 2007), Nipah virus (NiV) (Patch et al., 2007), HPIV3 (Zhang et al., 2014), and VSV (Li et al., 1993; Justice et al., 1995), expression of the M protein alone can direct budding and formation of VLPs. However, in the case of paramyxovirus simian virus 5 (SV5) M protein, NP and fusion protein (F) or hemagglutinin-neuraminidase (HN) are required for the formation and release VLPs (Schmitt et al., 2002). VP40 is the major matrix protein of EBOV; when expressed alone, it can be released into the culture medium as a type of VLP (Jasenosky et al., 2001; Timmins et al., 2001).

The M proteins of many NNSVs can regulate viral replication and/or transcription. The purified M protein of VSV condenses the nucleocapsid into a tight structure causing in vitro inhibition of viral transcription (De et al., 1982). The M protein of MeV retains the RNP complex at the plasma membrane by interacting with NP, thus inhibiting RNA synthesis and facilitating viral assembly and budding (Iwasaki et al., 2009). The M protein of HPIV3 suppresses the formation of IBs by interacting with NP, thereby reducing viral replication (Zhang et al., 2018). A previous study showed that VP40 inhibits replication and transcription of the Ebola minigenome (Hoenen et al., 2010). However, the detailed mechanism by which VP40 regulates viral RNA synthesis and NC assembly/budding remains largely unknown. In addition, from the perspective of virus evolution, in EBOV infected cells, when the virus synthesizes enough elements (RNAs and proteins) for packaging, the virus should shut down the synthesis of RNAs and proteins, and concentrate on the assembly/budding of virus particles to achieve the purpose of virion amplification. However, the molecular mechanism of how viruses transform from RNA synthesis to viral particle assembly is not clear.

In this study, we show that VP40 is first recruited to IBs by association with the N-terminus of NP, which results in a conformational change and exposure of the hydrophobic core within the NP C-terminus consisting of L692, P697, P698, and W699. Consequently, the exposed hydrophobic core within the NP C-terminus interacts with VP40 for the incorporation of NC-like structures into the VLPs of VP40. When the L692, P697, P698, and W699 within the hydrophobic core are mutated into alanines, stronger VP40–NP interactions occur, and the budding of VP40 is inhibited by sequestration of VP40 into IBs. Furthermore, we found that the hydrophobic core is critical for NP encapsidating viral RNA, and VP40 inhibits viral replication and transcription by association with this domain. These results suggest that the two-stage interaction of VP40 with NP plays a critical role in the transition from RNA synthesis to NC assembly/budding.

## Results

### N-terminal amino acids 26 to 150 of the NP are required for interactions with VP40 and for NP incorporation into VP40–VLP

Given the essential role played by NP and VP40 in EBOV RNA synthesis and NC assembly/budding, we first sought to study the relationship between VP40 and NP. We performed co-immunoprecipitation (coIP) and VLP budding assays, and the results showed that VP40 can interact with NP and incorporate NP into VP40–VLP, which was confirmed by the protease protection assay (Fig. S1A–C).

**Figure 1:**
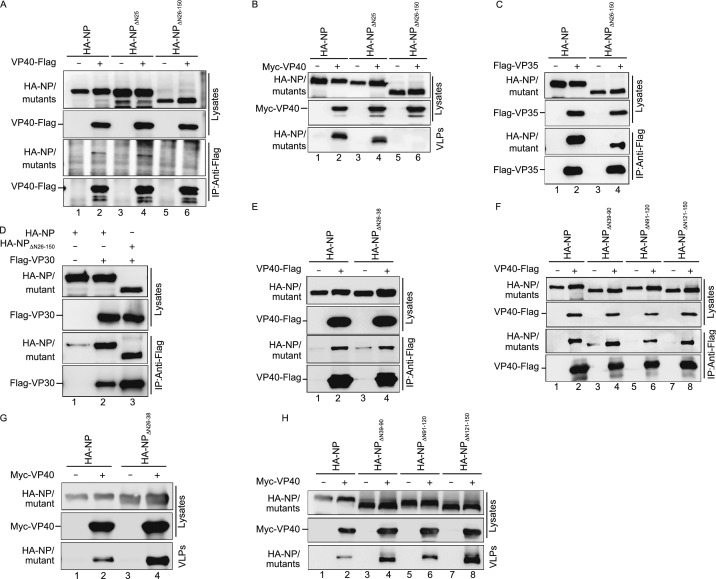
**N-terminal amino acids 26 to 150 of the NP are required for interactions with VP40 and for NP incorporation into VP40–VLP**. (A) HEK293T cells were co-transfected with HA-NP/NP N-terminal mutants and VP40-Flag. At 30 h p.t, lysates were subjected to IP with anti-Flag antibodies and analyzed via Western blot (WB). (B) HEK293T cells were co-transfected with HA-NP/NP N-terminal mutants and Myc-VP40 for 36 h. Cell lysates and VLPs from supernatants were subjected to WB with anti-Myc and anti-HA antibodies. (C and D) HEK293T cells were co-transfected with HA-NP/NP_∆N26–150_ and Flag-VP35 or Flag-VP30. Lysates were subjected to IP with anti-Flag antibodies and analyzed via WB. (C) The coIP of VP35 with NP_∆N26–150_; (D) the coIP of VP30 with NP_∆N26–150_. (E and F) HEK293T cells were transfected with the indicated plasmids. Lysates were subjected to IP with anti-Flag antibodies and analyzed via WB. (E) The coIP of VP40 with NP_∆N26–38_; (F) the coIP of VP40 with NP_∆N39–90_, NP_∆N91–120_, or NP_∆N121–150_. (G and H) HEK293T cells were transfected with the indicated plasmids. Cell lysates and VLPs from supernatants were subjected to WB. (G) The VLP budding assay of VP40 with NP_∆N26–38_; (H) the VLP budding assay of VP40 with NP_∆N39–90_, NP_∆N91–120_, or NP_∆N121–150_

A previous study showed that both the N- and C-termini of NP are important for interaction with VP40, and a 150 aa region within the NP N-terminus was found to be responsible for mediating the interaction with VP40 (Noda et al., 2007b). Based on this result, we sought to narrow down the specific amino acids within this region responsible for interaction, and generated two NP truncated mutants with an HA tag at the N-terminus (Leung et al., 2015; Su et al., 2018). We found that HA-NP_∆N25_ can interact with VP40-Flag as efficiently as WT NP, whereas the mutant HA-NP_∆N26–150_ is barely co-immunoprecipitated by VP40-Flag (Fig. 1A, lanes 2, 4, and 6). Meanwhile, Myc-VP40 can incorporate HA-NP_∆N25_ but not HA-NP_∆N26–150_ into VLPs, which is consistent with the coIP result (Fig. 1B, bottom panel, lanes 4 and 6). In addition, we observed that NP_∆N26–150_ still maintains its associations with VP35 and VP30 (Fig. 1C, lane 4 and 1D, lane 3), which excludes the possibility that the structure of NP_∆N26–150_ has been changed and can no longer be recognized by VP40. Next, to further narrow down the region within the N-terminus of NP involved in the interaction with VP40, we constructed a series of NP N-terminal truncated mutants based on a previous study (Sugita et al., 2018). We found that all the mutants with shorter truncations could be co-immunoprecipitated and incorporated into VLP s by VP40 (Fig. 1E–H), suggesting that more than one domain within amino acids 26 to 150 in the NP N-terminus is involved in the association of NP–VP40 and the incorporation of NP into VP40–VLP.

Because NP expression alone can induce the formation of IBs, we sought to examine whether the expression of the above NP N-terminal mutants can also form IBs. Toward this aim, HeLa cells were transfected with NP or N-terminal mutants. We also observed that the mutants NP_∆N25_ and NP_∆N26–38_ could aggregate in the cytoplasm, similar to those formed by NP. However, the mutants NP_∆N39–90_, NP_∆N91–120_, and NP_∆N121–150_ were all homogeneously distributed in the cytoplasm (Fig. S1D). To determine whether the failure of NP mutants to form IBs is related to their oligomerization, we performed a coIP assay. As expected, HA-NP_∆N25_ and HA-NP_∆N26–38_ interacted with Myc-NP, while HA-NP_∆N39–90_, HA-NP_∆N91–120_, and HA-NP_∆N121–150_ failed to interact with Myc-NP (Fig. S1E–F), suggesting that amino acids 39 to 150 in the N-terminus are critical for the formation of IBs and the oligomerization of NP.

Taken together, our results demonstrate that N-terminal amino acids 26 to 150 are critical for the interaction of VP40 and NP as well as the incorporation of NP into VP40–VLP.

### **Incorporation of NP into VP40–VLP via the NP C**-**terminal hydrophobic core**

**Figure 2:**
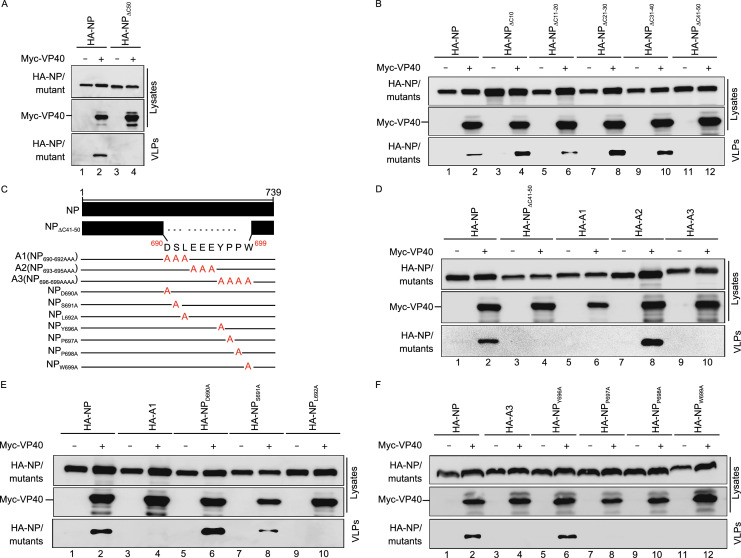

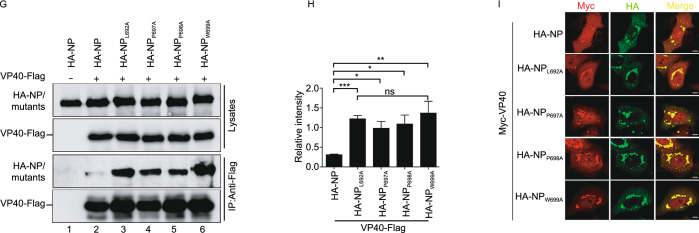
**Incorporation of NP into VP40–VLP via the NP C-terminal hydrophobic core**. (A) HEK293T cells were co-transfected with HA-NP/NP_∆C50_ and Myc-VP40 for 36 h. Cell lysates and VLPs from supernatants were subjected to WB analysis. (B) VLP budding assay for the co-transfection of HA-NP/NP C-terminal truncated mutants with Myc-VP40. HEK293T cells were transfected with the indicated plasmids for 36 h. Cell lysates and VLPs were analyzed via WB. (C) Schematic diagrams of C-terminally truncated or point mutants of NP. (D–F) VLP budding assay of HA-NP/NP C-terminal point mutants with Myc-VP40. HEK293T cells were transfected with the indicated plasmids for 36 h. Cell lysates and VLPs were analyzed via WB. (G) coIP assay of the VP40-Flag with HA-NP/NP_L692A_, NP_P697A_, NP_P698A_, or NP_W699A_. HEK293T cells were transfected with the indicated plasmids. At 30 h p.t, cell lysates were subjected to IP with anti-Flag antibodies and then analyzed via WB. (H) HEK293T cells were treated as above. The interactions of HA-NP/NP_L692A_, NP_P697A_, NP_P698A_, or NP_W699A_ with VP40-Flag were quantified using the Quantity One software. (I) The colocalization of VP40 with NP/NP_L692A_, NP_P697A_, NP_P698A_, or NP_W699A_. HeLa cells were co-transfected with Myc-VP40 and HA-NP/NP mutants for 26 h. Cells were immunostained for VP40 (red) and NP or NP mutants (green) and analyzed using superresolution microscopy (Zeiss LSM 800). Scale bars = 5 μm. Images are representative of three independent experiments. Error bars, mean ± SD of three experiments (*n* = 3). Student’s *t*-test; *P < 0.05; **P < 0.01; ***P < 0.001; ns, non-significant

We thus established that the NP N-terminus is required for the incorporation of NP into VP40–VLP. In addition, a previous study showed that NP C-terminal 50 amino acids are critical for NP’s incorporation into VP40–VLP (Licata et al., 2004). Next, we sought to determine how the N- and C-termini of NP participate in the incorporation of NP into VP40–VLP. Using a VLP budding assay, we first confirmed that a truncated NP whereby the last 50 amino acids in the C-terminus were deleted (NP_∆C50_) failed to incorporate NP into VP40–VLP (Fig. 2A, bottom panel, lane 4). Moreover, we found that NP_∆C50_ still maintains the associations with VP35 and VP30 (Fig. S2A and S2B, lanes 4, 3), suggesting that the structure of NP_∆C50_ is intact and undamaged. Then, to narrow down the specific amino acids within this 50 amino acid region responsible for incorporation of NP into VP40–VLP, we generated five other mutants by sequential deletion, whereby each contained 10 amino acids as shown in Fig. S2C, and found that for the same level of expression, NP_∆C41–50_ clearly lost the ability to be incorporated into VP40–VLP (Fig. 2B, bottom panel, lane 12), suggesting that amino acids 690 to 699, located at the NP C-terminus, are critical for the incorporation of NP into VP40–VLP (Fig. 2C). To further explore the critical motif for NP incorporation into VP40–VLP in amino acids 690 to 699, we constructed three different triple or quadruple-point mutants A1 (NP_690–692AAA_), A2 (NP_693–695AAA_), and A3(NP_696–699AAAA_) (Fig. 2C) and found that neither A1 nor A3 could be incorporated into VP40–VLP, in contrast to A2 (Fig. 2D, lanes 6, 8, and 10). To determine the precise binding site(s), we generated seven point mutants containing unique alanine mutations within A1 and A3 (Fig. 2C), and these mutants were used for the VLP assay. We found that, similar to A1 and A3, four mutants (NP_L692A_, NP_P697A_, NP_P698A_, and NP_W699A_) could not be incorporated into VP40–VLP (Fig. 2E, lanes 4, 10 and 2F, lanes 4, 8, 10 and 12). Sequence alignments show that L692, P697, P698, and W699 are relatively conserved among the filovirus family (Fig. S2D) and exist in NP as side chains which constitute a hydrophobic core, according to an analysis of NP C-terminal structure (PDB: 4QB0) (Fig. S2E). Taken together, these results show that four sites (L692, P697, P698, and W699) of the hydrophobic core in the NP C-terminus are indispensable for incorporation of NP into VP40–VLP.

Because NP–VP40 interactions are a prerequisite for NP incorporation into VP40–VLP, and having found that NP_L692A_, NP_P697A_, NP_P698A_, and NP_W699A_ cannot be incorporated into VP40–VLP, we sought to determine whether these mutants fail to interact with VP40. However, to our surprise, we found that all these mutants significantly enhanced interactions with VP40 compared to WT NP at the same level of expression (Fig. 2G–H). A previous study showed that VP40 can be recruited into IBs in EBOV-infected cells (Nanbo et al., 2013). Thus, we sought to determine whether the expression of these mutants could form IBs, and if so, whether the IBs formed by these mutants could recruit VP40. The results showed that the IBs formed by the expression of NP_L692A_, NP_P697A_, NP_P698A_, and NP_W699A_ are similar in size and morphology to those of WT NP (Fig. S2F). When VP40 was expressed alone, it was homogeneously distributed in the cytoplasm and nucleus, and we observed a filamentous structure on the plasma membrane (Fig. S2G). We also found that VP40 was, indeed, recruited into the IBs but did not change the size and morphology of the IBs when co-expressed with WT NP. However, when VP40 was co-expressed with NP_L692A_, NP_P697A_, NP_P698A_, or NP_W699A_, VP40 was not only recruited into IBs but also resulted in the aggregation of IBs which were significantly larger in size than those formed by the co-expression of NP and VP40 (Fig. 2I). VP35 or VP30 could also colocalize with IBs but had no effect on the size and morphology of the IBs formed by NP_L692A_, NP_P697A_, NP_P698A_, or NP_W699A_ (Fig. S2H and S2I). Taken together, these results show that both the N- and C-termini of NP are required for the incorporation of NP into VP40–VLP.

### NP_L692A_, NP_P697A_, NP_P698A_, and NP_W699A_ inhibit the release of VP40–VLP

**Figure 3:**
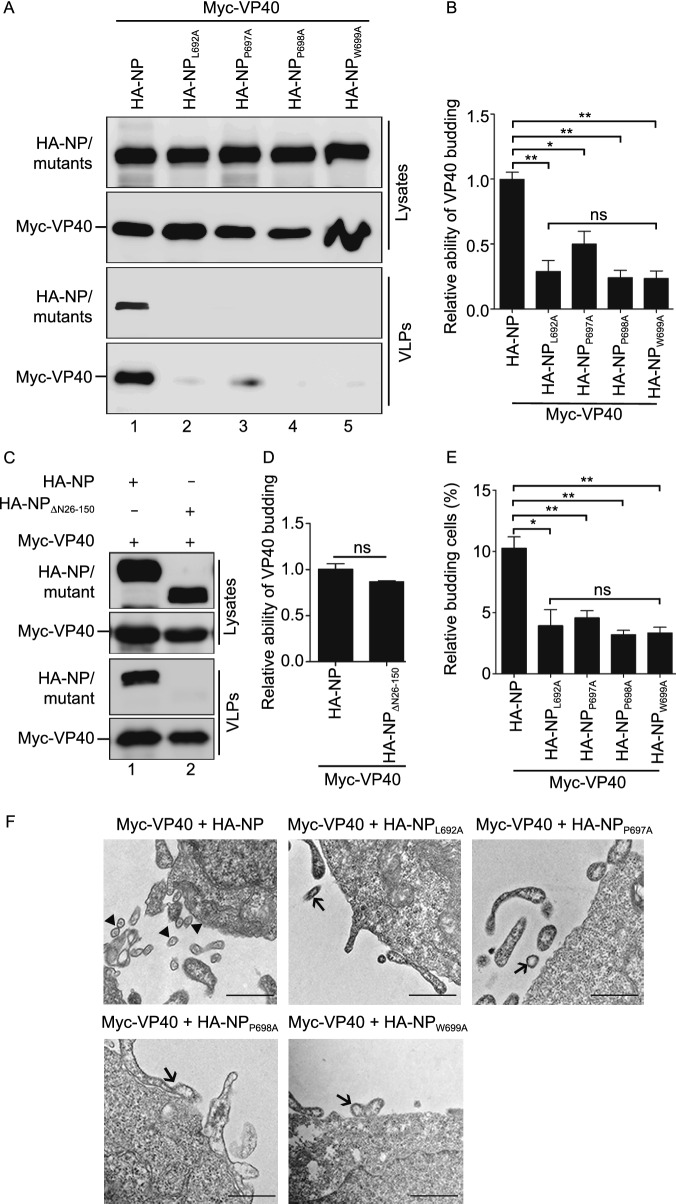
**NP**_**L692A**_**, NP**_**P697A**_**, NP**_**P698A**_**, and NP**_**W699A**_ **inhibit the release of VP40–VLP**. (A and B) VLP budding assay of VP40 in the presence of NP/NP_L692A_, NP_P697A_, NP_P698A_, or NP_W699A_. HEK293T cells were co-transfected with Myc-VP40 and HA-NP/NP mutants for 36 h. The VLP budding assay was performed as mentioned in Fig. 2A (A); the ability of VP40–VLP to be released was quantified using Quantity One software (B). (C and D) VLP budding assay of VP40 in the presence of NP/NP_∆N26–150_. HEK293T cells were co-transfected with Myc-VP40 and HA-NP/NP_∆N26–150_ for 36 h. (C) The VLP budding assay was performed as mentioned in Fig. 2A; (D) the ability of VP40–VLP egress was quantified using Quantity One software. (E) Budding cells under VP40 co-transfection with NP/NP_L692A_, NP_P697A_, NP_P698A_, or NP_W699A_. HeLa cells were treated as mentioned in Fig. 2I. The cells with filamentous structures were quantified (*n* = 3, 100 cells were counted). Images are representative of three independent experiments. Error bars, mean ± SD of three experiments (*n* = 3). Student’s *t*-test; *P < 0.05; **P < 0.01; ***P < 0.001; ns, non-significant. (F) Budding assay of VP40 via transmission electron microscopy. HEK293T cells were transfected with the indicated plasmids for 36 h. Budding cells were observed via electron microscopy. Black triangles indicate VLPs containing NP; black arrows indicate empty VP40–VLP. Scale bars = 500 nm

Since VP40 has stronger interactions with NP_L692A_, NP_P697A_, NP_P698A_, and NP_W699A_ and induces the aggregation of IBs but fails to incorporate these mutants into VLPs, we hypothesized that the interaction of the NP N-terminus with VP40 is a prerequisite for the incorporation of NP into VP40–VLP and only contributes to recruiting VP40 into IBs. If only the N-terminal interaction with VP40 is preserved, and the C-terminal interaction of NP with VP40 is deprived, VP40 will be trapped in the IBs, thereby inhibiting the assembly/release of VP40–VLP. To test this, we co-expressed VP40 with NP_L692A_, NP_P697A_, NP_P698A_, or NP_W699A_ and performed a VLP budding assay. As expected, VP40 could be efficiently detected in the VLPs when co-expressed with NP. However, the release of VP40–VLP was reduced by 2–4 fold in the case of NP_L692A_, NP_P697A_, NP_P698A_, or NP_W699A_ (Fig. 3A and 3B). NP_∆N26–150_, however, no longer interacted with VP40 and, therefore, had no effect on the release of VP40–VLP (Fig. 3C and 3D). Furthermore, we found that the numbers of cells containing filamentous structures in VP40-expressing cells were dramatically decreased in the presence of NP_L692A_, NP_P697A_, NP_P698A_, or NP_W699A_ (Fig. 3E), suggesting that more VP40 is sequestered in the IBs formed by NP_L692A_, NP_P697A_, NP_P698A_, or NP_W699A_, thereby inhibiting the release of VP40–VLP. Furthermore, by using transmission electron microscopy, we observed that many VP40–VLP containing NP are located around the plasma membrane (PM), but that fewer empty VP40–VLP could be observed around the PM when VP40 was co-expressed with NP_L692A_, NP_P697A_, NP_P698A_, or NP_W699A_ (Fig. 3F). Taken together, our results show that four sites, L692, P697, P698, and W699, located in the hydrophobic core of the C-terminus of NP, are indispensable for the assembly/budding of NP–VP40 VLPs, while NP_L692A_, NP_P697A_, NP_P698A_ and NP_W699A_ inhibit the release of VP40–VLP by sequestering VP40 into IBs.

### NP_L692A_, NP_P697A_, NP_P698A_, and NP_W699A_ inhibit the migration of VP40 to the plasma membrane

**Figure 4:**
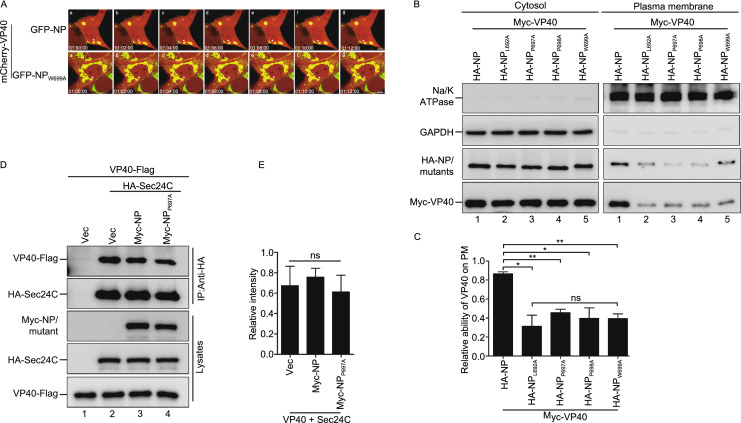
**NP**_**L692A**_**, NP**_**P697A**_**, NP**_**P698A**_**, and NP**_**W699A**_ **inhibit the migration of VP40 to the plasma membrane**. (A) The kinetic progress of IB migration to the PM. HeLa cells were cultured on 20 mm dishes and co-transfected with mCherry-VP40 and GFP-NP or GFP-NP_W699A_, together with their respective functional proteins, to exclude the effect of fluorescence tags. Live-cell imaging analysis was started at 24 h post-transfection. The pictures show the maximum intensity projection of time-lapse images of the cells. Images were captured every 120 s. Scale bar = 5 μm. (B and C) Isolation of cytosol and plasma membrane (PM) fractions. HEK293T cells were transfected with the indicated plasmids for 24 h. The cytosol and PM fractions were isolated and then subjected to a WB assay. GAPDH and Na/K ATPase served as controls for the cytosol or the PM fraction, respectively (B); quantification of the amount of PM-associated VP40 is shown (C). (D and E) The impact of NP_P697A_ on the interaction of VP40 with Sec24C. HEK293T cells were co-transfected with VP40-Flag, HA-Sec24C, and Myc-NP/NP_P697A_ for 30 h. IP was performed with anti-HA antibodies (D); quantification of the ability of VP40 binding Sec24C in the presence of NP/NP_P697A_ is shown (E). Images are representative of three independent experiments. Error bars, mean ± SD of three independent experiments (*n* = 3). Student’s *t*-test; *P < 0.05; **P < 0.01; ***P < 0.001; ns, non-significant

The efficient egress of VP40 requires its transportation to the PM, where it finishes self-assembly/budding (Bornholdt et al., 2013). Since NP_L692A_, NP_P697A_, NP_P698A_, or NP_W699A_ sequester VP40 into IBs, we hypothesized that alterations in VP40 localization would correlate with budding inhibition. To test this, live-cell imaging was performed. For this, we co-expressed mCherry-VP40 and GFP-NP or GFP-NP_W699A_ in HeLa cells, and found that VP40 colocalized with IBs formed by NP; these IBs were able to co-migrate to the PM. Although VP40 could also colocalize with the IBs induced by NP_W699A_, their ability to migrate to the PM was inhibited (Fig. 4A; Video. S1A and S1B). Furthermore, we utilized a biochemical approach to determine whether the levels of VP40 in the PM fraction could be reduced in the presence of NP_L692A_, NP_P697A_, NP_P698A_, or NP_W699A_. For this, HEK293T cells were transfected with either VP40 and NP, or VP40 in combination with one of these mutants, and we found that when normalized to equivalent levels in the cytoplasm, the levels of VP40 in the PM fraction were reduced by 2–3 fold in the presence of NP_L692A_, NP_P697A_, NP_P698A_, or NP_W699A_ compared to the levels in the presence of NP (Fig. 4B and 4C). Taken together, these results show that NP_L692A_, NP_P697A_, NP_P698A_, and NP_W699A_ inhibit the migration of VP40 migration to the PM by sequestering VP40 into IBs.

A previous study demonstrated that VP40 can interact with Sec24C, a component of the COPII transport system which is critical for the intracellular transport of VP40 to the PM (Yamayoshi et al., 2008). Therefore, we sought to determine whether these mutants affect VP40–Sec24C interaction. We chose NP_P697A_ and performed a coIP assay and found that NP_P697A_ had no effect on VP40–Sec24C interaction (Fig. 4D, upper panel, lanes 2, 4 and 4E), suggesting that the NP–VP40 complex may use a different mechanism for transport, which requires further investigation.

### **The NP C**-**terminal hydrophobic core is critical for the incorporation of nucleocapsid**-**like structures into VP40**–**VLP**

**Figure 5:**
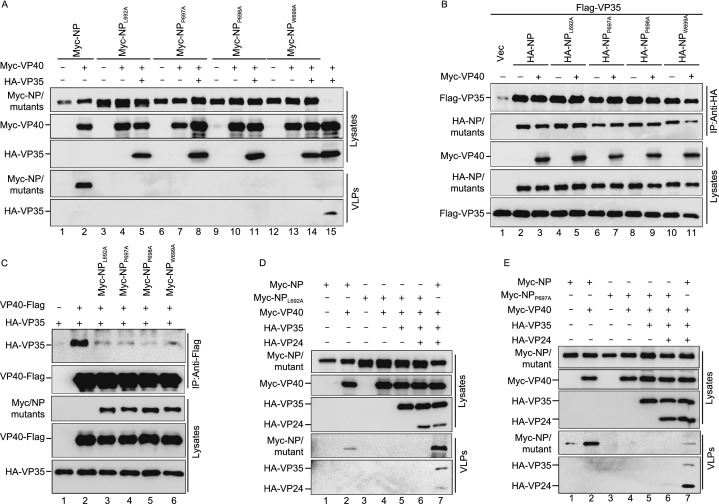

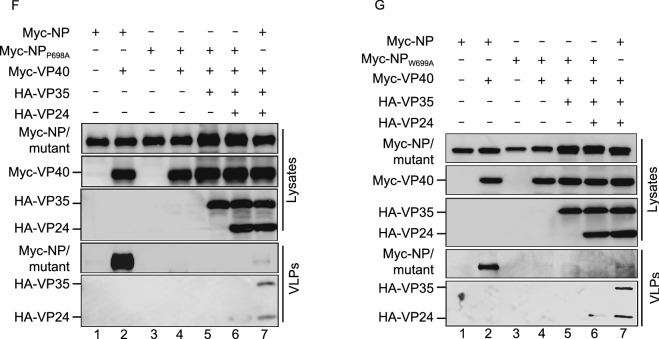
**The NP C-terminal hydrophobic core is critical for the incorporation of nucleocapsid-like structures into VP40–VLP**. (A) VP40 assembles the NP–VP35 complex into VLPs via the NP C-terminal hydrophobic core. HEK293T cells were transfected with the indicated plasmids. Cell lysates and VLPs from the supernatants were subjected to WB assay with anti-Myc and anti-HA antibodies. (B) The effect of VP40 on the interactions of NP_L692A_, NP_P697A_, NP_P698A_, or NP_W699A_ with VP35. HEK293T cells were transfected with the indicated plasmids, and IP was performed with the anti-HA antibodies. WB was performed with anti-Flag, anti-HA, and anti-Myc antibodies. (C) The effect of NP_L692A_, NP_P697A_, NP_P698A_, or NP_W699A_ on the interaction of VP40 with VP35. HEK293T cells were transfected with the indicated plasmids. Cell lysates were subjected to IP with anti-Flag antibodies and analyzed via WB. (D–G) The association of the NP C-terminal hydrophobic core with VP40 mediates the NC-like structures assembly. NP or one of the C-terminal mutants with VP40, VP35, and VP24 were co-transfected into HEK293T cells. Cell lysates and VLPs from the supernatants were subjected to a WB assay with anti-Myc and anti-HA antibodies in the presence of NP_L692A_ (D); in the presence of NP_P697A_ (E); in the presence of NP_P698A_ (F); and in the presence of NP_W699A_ (G)

We found that VP40 incorporated NP into VLPs via the NP C-terminal hydrophobic core, and a previous study also showed that VP35 could be incorporated into VP40–VLP (Johnson et al., 2006). Therefore, we sought to determine what would happen when NP, VP35, and VP40 were co-expressed. We found that NP or VP35 could be efficiently incorporated into VP40–VLP (Fig. 5A, lanes 2 and 15), while the NP C-terminal mutants NP_L692A_, NP_P697A_, NP_P698A_, and NP_W699A_ could not be incorporated into VP40–VLP in the presence of VP35. Furthermore, the incorporation of VP35 into VP40–VLP was also severely inhibited by NP_L692A_, NP_P697A_, NP_P698A_, and NP_W699A_ (Fig. 5A, lanes 5, 8, 11 and 14), suggesting that the assembly of the NP–VP35 complex into VP40–VLP is mediated via the association of the NP hydrophobic core with VP40, rather than VP35. To confirm this, we performed coIP assay and found that NP_L692A_, NP_P697A_, NP_P698A_, and NP_W699A_ interacted with VP35 as efficiently as NP (Fig. 5B, upper panel, lanes 2, 4, 6, 8, and 10). Moreover, VP40 did not affect the interactions of NP_L692A_, NP_P697A_, NP_P698A_, or NP_W699A_ with VP35 (Fig. 5B, upper panel, lanes 4 to 11), but NP_L692A_, NP_P697A_, NP_P698A_, and NP_W699A_ significantly inhibited the interaction of VP35 with VP40 (Fig. 5C, upper panel, lanes 2 to 6), suggesting that NP–VP35 complex is incorporated into VP40–VLP via NP–VP40 interaction.

It has also been established that NP, VP35, and VP24 are the minimal requirements for the formation of NC-like structures of EBOV (Huang et al., 2002). Moreover, we found that VP24 can interact with VP40 and be incorporated into VLPs (Fig. S3A and S3B, lanes 2). Therefore, we sought to determine how VP40 assembles NC-like structures. For this, we expressed NP or one of the NP C-terminal mutants, VP35 and VP24, with VP40 and then performed VLP budding assays. The results show that VP40 could indeed incorporate the NP–VP35–VP24 complex into VLPs (Fig. 5D–G, lanes 7). However, the complex formed by one of the NP C-terminal mutants, VP35 and VP24, was not able to be incorporated into the VLPs (Fig. 5D–G, lanes 6). Taken together, these results show that the association of the NP C-terminal hydrophobic core with VP40 mediates assembly of the NC-like structures.

### **Interaction of VP40 with the NP C**-**terminal hydrophobic core prevents NP from encapsidating viral RNA**

**Figure 6:**
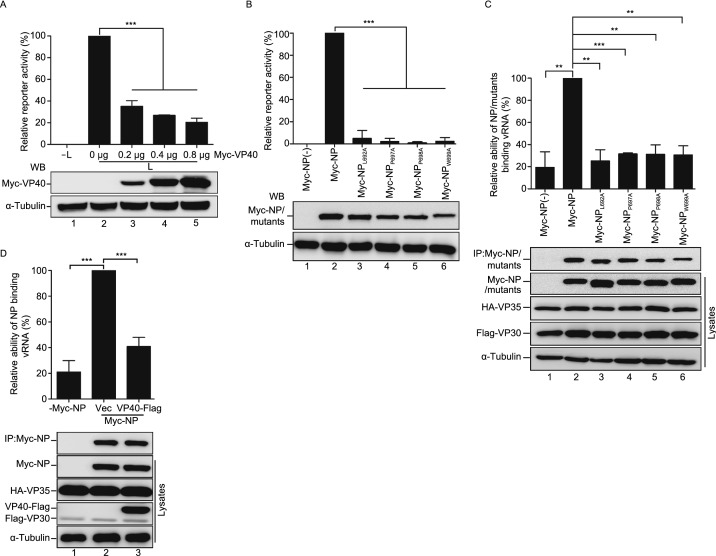
**Interaction of VP40 with the NP C-terminal hydrophobic core prevents NP from encapsidating viral RNA**. (A) The effect of VP40 on Ebola minigenome RNA synthesis activity. HEK293T cells were co-transfected with plasmids encoding the minigenome assay components (NP, VP35, VP30, L, pBS-Rluc, Luc2, and T7) and increasing amounts of VP40. *Renilla* luciferase activity normalized to the firefly luciferase values was measured following the manufacturer’s instructions. The expression of VP40 was analyzed via WB. (B) The minigenome RNA synthesis ability of NP_L692A_, NP_P697A_, NP_P698A_, or NP_W699A_. Plasmids encoding the minigenome assay components (VP35, VP30, L, pBS-Rluc, T7, and Luc2) and Myc-NP/NP C-terminal point mutations were co-transfected into HEK293T cells. *Renilla* luciferase activity normalized to the firefly luciferase values was measured following the manufacturer’s instructions. The expression of Myc-NP and NP mutants was detected via WB. (C) The ability of NP_L692A_, NP_P697A_, NP_P698A_, or NP_W699A_ binding minigenome RNA. Plasmids encoding the minigenome assay components (VP35, VP30, L, pBS-Rluc, and T7) and Myc-NP/NP C-terminal point mutations were co-transfected into HEK293T cells. Cell lysates were subjected to IP with anti-Myc antibodies, and then RNA was extracted, followed by RT-qPCR. The data are represented as relative scales, with the IP minigenome RNA normalized against the cell lysate’s minigenome RNA for each sample. The values were expressed as a percentage, where 100% was taken as abundance of immunoprecipitated Myc-NP. IP and cell lysate fractions were analyzed via WB. (D) VP40 inhibits NP binding the minigenome RNA. Plasmids encoding the minigenome assay components (Myc-NP, VP35, VP30, L, pBS-Rluc, and T7) and VP40-Flag were co-transfected into HEK293T cells. Cell lysates were subjected to IP with anti-Myc antibodies, and then RNA was extracted, followed by RT-qRCR. The data are represented as relative scales, with the IP minigenome RNA normalized against the cell lysate’s minigenome RNA for each sample. The values were expressed as a percentage, where 100% was taken as abundance of immunoprecipitated Myc-NP. IP and cell lysate fractions were analyzed via WB. Images are representative of three independent experiments. Error bars, mean ± SD of three independent experiments (*n* = 3). Student’s *t*-test; *P < 0.05; **P < 0.01; ***P < 0.001; ns, non-significant

We established that the NP C-terminal hydrophobic core plays an essential role in the incorporation of NC-like structures into VP40–VLP. In addition, it was also reported that VP40 could regulate viral RNA synthesis (Hoenen et al., 2010), but the molecular mechanism through which VP40 regulates viral RNA is unclear. Therefore, we hypothesized that the hydrophobic core in the NP C-terminus may play an essential role in viral RNA synthesis and that its association with VP40 may inhibit viral RNA synthesis, thus causing a switch from viral RNA synthesis to virion assembly. To confirm this, we first performed an Ebola minigenome assay with increasing amounts of VP40 and found that VP40 inhibits viral RNA synthesis in a dose-dependent manner (Fig. 6A). Then, we further examined the RNA synthesis activity of NP_L692A_, NP_P697A_, NP_P698A_, and NP_W699A_ and found that all four mutants barely support minigenome-encoded *Renilla* luciferase expression (Fig. 6B), suggesting that the NP C-terminal hydrophobic core is, indeed, indispensable for viral RNA synthesis.

Because the oligomerization of NP and NP–VP30 interaction are essential for the RNA synthesis function of NP (Kirchdoerfer et al., 2015; Leung et al., 2015; Kirchdoerfer et al., 2016; Xu et al., 2017; Batra et al., 2018; Sugita et al., 2018), we sought to determine whether these mutants disrupt the oligomerization of NP or NP–VP30 interaction. We found that all these mutants can interact with NP or VP30 as efficiently as NP (Fig. S4A and S4B), which is consistent with the immunofluorometric assay results (Fig. S2F and S2I). In addition, a previous study showed that NP acts as a bridge to recruit PP2A-B56 via the LxxIxE motif for VP30 dephosphorylation, thus activating viral transcription (Xu et al., 2006; Hertz et al., 2016; Kruse et al., 2018). To further determine whether the NP C-terminal mutants are still able to dephosphorylate VP30, we co-expressed VP30 with NP or NP_P697A_ and found that the levels of VP30 Ser29 phosphorylation were similarly reduced by NP_P697A_, comparable to that in the presence of NP (Fig. S4C). Taken together, these results showed that the NP C-terminal mutants maintain their normal functions of oligomerization, interactions with other nucleocapsid proteins, and VP30 dephosphorylation.

Next, we sought to determine whether these mutants affect NP encapsidation of viral RNA. To test this, we performed an immunoprecipitation-RT-qPCR assay using the Ebola virus minigenome system and found that amount of immunoprecipitated minigenome RNA was severely reduced for the NP C-terminal mutants compared to NP (Fig. 6C), suggesting that the NP C-terminal hydrophobic core is essential for NP encapsidation of viral RNA. To determine how VP40 inhibits viral RNA synthesis, we examined the effects of VP40 on NP oligomerization and NP–VP30 or VP35–VP30 interactions. We found that VP40 had no effect on NP oligomerization, NP–VP30 or VP35–VP30 interactions (Fig. S4D–F), suggesting that VP40 did not affect the formation of NCs. However, as expected, we found that the ability of NP binding minigenome RNA was dramatically reduced in the presence of VP40 (Fig. 6D), suggesting that the interaction of VP40 with the NP C-terminal hydrophobic core prevents NP from encapsidating viral RNA, thus inhibiting viral RNA synthesis.

## Discussion

In a previous study, both the N- and C-termini of NP were found to be important for the interaction of NP with VP40 and the incorporation of NP into VP40–VLP (Noda et al., 2007b). However, how both ends regulate the incorporation of NP into VP40–VLP remains unclear. Here, we show that VP40 is first recruited into IBs by interacting with the NP N-terminus, which subsequently results in conformational change of the NP C-terminus and exposure of the hydrophobic core that associates with VP40 for the incorporation of NP into VLPs. This conclusion is supported by the following evidence. First, the NP N-terminal mutant, NP_∆N26–150_, failed to interact with VP40 and prevented the incorporation of NP into VP40–VLP (Fig. 1A and 1B). In addition, the structural analysis of the NP and VP40 interacting regions was performed using the HDOCK server based on NP core structure (PDB: 4Z9P) and the VP40 structure (PDB: 1ES6) (Fig. S5A). The top three predicted results were analyzed and we found that the NP-VP40 model showed that with the third highest docking score exhibited the largest interacting area of the three (Fig. S5B), while the top two models had larger interacting region with the C-lobe (Fig. S5C–D). In addition, previous co-IP result showed that the region from amino acid 151 to 300 (within the C-lobe) of NP is dispensable for VP40 binding (Noda et al., 2007b). Taken together, the docking analysis was consistent with our functional data. Moreover, NP_∆26–150_ maintained its interactions with VP35 and VP30 (Fig. 1C and 1D), indicating that the C-terminal structure of NP_∆26–150_ is similar to that of NP, which also indicates that the intact C-terminus of NP is unable to interact with VP40. Second, we identified four key amino acids (L692, P697, P698, and W699) at the NP C-terminus which form the hydrophobic core (Fig. S2E); when mutated to alanines (NP_L692A_, NP_P697A_, NP_P698A_, and NP_W699A_) the incorporation of NP into VP40–VLP was abolished but the interaction with VP40 became stronger (Fig. 2E–H). Furthermore, we also observed that VP40 colocalized with the IBs formed by these mutants, resulting in the aggregation of IBs (Fig. 2I). Third, the release of VP40–VLP was also inhibited by NP_L692A_, NP_P697A_, NP_P698A_, and NP_W699A_ (Fig. 3A, 3B, 3E and 3F), similar to a previous study that showed the final 50 amino acids in the NP C-terminus regulate the budding of VP40 (Licata et al., 2004), suggesting that any mutation within L692, P697, P698, and W699 would prevent conformational changes in the hydrophobic core resulting from interactions of the NP N-terminus with VP40. On the one hand, this results in the hydrophobic core of NP C-terminus being unable to expose itself and interact with VP40; on the other hand, the N-terminal interaction with VP40 is preserved, thereby sequestering VP40 in IBs and causing failure of VP40–VLP budding.

We found that NP_P697A_ had no effect on the interaction of VP40 with Sec24C (Fig. 4D and 4E), suggesting NP–VP40 uses a different mechanism for cellular transport. A previous study revealed that EBOV nucleocapsids are dependent on actin for long-distance transport in Ebola virus-infected cells (Schudt et al., 2015), which indicates that mutations in the NP C-terminus may affect the interaction of VP40 with cytoskeleton proteins, thus inhibiting their cellular transport.

We also found that the NP C-terminal hydrophobic core is critical for NC-like structures to be recruited to viral particles (Fig. 5). Similar to these results, our previous result showed that the interaction of NP with the M protein of HPIV3 regulates the NC-like structures assembly (Zhang et al., 2015). Moreover, the NP C-terminus of HPIV1 and SeV was also reported to mediate interaction with M protein, which is critical for virion incorporation (Coronel et al., 2001). The NP of the influenza virus is essential for virion assembly, possibly via association with M1 (Noton et al., 2009). However, for RSV, the transcriptional antiterminator M2-1 acts as a bridge in linking RNPs with M protein, which is required for the incorporation of RNPs into virions (Li et al., 2008). These results suggest that the mechanisms by which RNPs are assembled and incorporated into viral particles seem to differ among the different viruses. In addition, we could not exclude the possibility that VP35 and VP24 play a role in the correct assembly of virions (Bharat et al., 2012). We also need to further investigate the regions within VP40 that regulate interaction with nucleocapsid proteins, which may provide essential insights into the mechanism underlying the incorporation of Ebola internal proteins into virions.

The M proteins of some NNSVs can regulate RNA synthesis. For EBOV, we confirmed that VP40 inhibits RNA synthesis in a dose-dependent manner (Fig. 6A). Different viruses utilize different mechanisms to regulate RNA synthesis. The M protein of HPIV3 and MeV inhibits RNA synthesis by interacting with NP and, especially in the case of MeV, it is possible that M protein inhibits RNA synthesis to promote viral particle production (Iwasaki et al., 2009; Zhang et al., 2018). Previous studies suggested that EBOV NP oligomerization and NP–RNA binding are cooperative and do not occur independently (Sugita et al., 2018). VP40 can be recruited into IBs via the NP N-terminus that is critical for NP oligomerization (Figs. S1D–F and 2). However, we found that VP40 has no effect on NP oligomerization (Fig. S4D). Moreover, the binding regions of RNA and VP40 at the NP N-terminus do not overlap (Dong et al., 2015; Sugita et al., 2018), making it unlikely that VP40 inhibits RNA synthesis by interacting with the NP N-terminus. Meanwhile, we found that VP40 has no effect on nucleocapsid formation (Fig. S4E and S4F).

Our results show that NP_L692A_, NP_P697A_, NP_P698A_, and NP_W699A_ have almost complete inhibition of RNA synthesis (Fig. 6B), suggesting that the hydrophobic core is indispensable for RNA synthesis. Moreover, we found that these mutants maintain the functions of NP in oligomerization, interacting with other nucleocapsid proteins and engaging in the dephosphorylation of VP30 (Figs. S4A–C and 5B). It is possible that the hydrophobic core could regulate NP encapsidation of viral RNA by changing the state of folding and unfolding, which was confirmed by an IP-RT-qPCR assay (Fig. 6C). Since the NP C-terminal hydrophobic core is critical both for viral RNA synthesis and NC assembly/budding, it was important to determine whether VP40 regulates RNA synthesis and NC assembly/budding by interacting with this domain. We also found that VP40 inhibits NP encapsidation of viral RNA (Fig. 6D). These results suggest that VP40 inhibits RNA synthesis to facilitate NC assembly/budding by interacting with the NP C-terminal hydrophobic core. In addition, we need to further investigate the domains within VP40 that regulate the interaction of VP40 with the NP C-terminal hydrophobic core. Meanwhile, we cannot exclude possibility that cellular factor(s) participate in these processes by interacting with either VP40 or NP, or both.

In the evolution of EBOV, the mRNAs of NP are firstly transcribed, and then go through translation for protein synthesis. Moreover, the amount of NP is much more than VP40 in the cytoplasm and NP preferentially encapsidates viral RNA to form NP-RNA template for RNAs and proteins synthesis. When elements (RNAs and proteins) for packaging are enough, the virus is prepared for shutting down the synthesis of RNAs and proteins, and focusing on the assembly/budding. At this point, VP40 is recruited into IBs via the interaction with NP N-terminus, and then their interaction is switched to NP C-terminus, thereby preventing NP from encapsidating of newly synthesized RNA, thus inhibiting RNA synthesis. However, once having formed NP-RNA template, VP40 interacts with NP in the same way, thus initiating nucleocapsids (NCs) assembly/budding. Therefore, we think that VP40 inhibiting RNA encapsidation to initiate viral assembly is not a paradox in terms of viral lifecycle.

**Figure 7:**
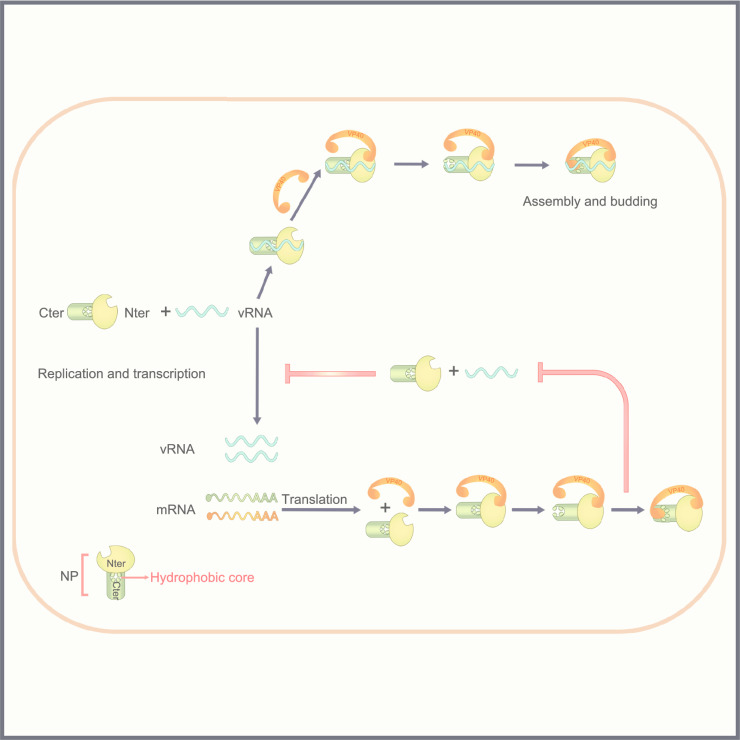
**Model of the two-stage interaction of VP40 with nucleoprotein resulting in a switch from viral RNA synthesis to virion assembly/budding**

In conclusion, we suggest a model (Fig. 7) through which VP40 can be recruited into IBs mediated by the NP N-terminus, with such interaction then resulting in a conformational change in the NP C-terminus and exposure of the hydrophobic core. On the one hand, when VP40 associates with the NP C-terminal hydrophobic core, VP40 inhibits RNA synthesis by preventing NP from encapsidating of newly synthesized viral RNA (viral RNA encapsidation did not occur); on the other hand, VP40 incorporates NC (viral RNA encapsidation occurred) into VLPs for assembly/budding of virion. To our best knowledge, this is the first study to demonstrate how a two-stage VP40–NP interaction plays a critical role in switching from viral RNA synthesis to assembly/ budding, which suggests that this interaction may be a promising target for antiviral therapy.

## Materials and methods

### Cell culture

HEK293T and HeLa cells were purchased from China Center for Type Culture Collection (CCTCC) and maintained in Dulbecco’s modified Eagle’s medium (DMEM, Gibco), supplemented with 15% or 8% fetal bovine serum (FBS, Gibco) at 37 °C with 5% CO_2._

### Plasmid construction

The cDNA of the *Zaire ebolavirus* and the expression plasmids pCAGGS-NP, pCAGGS-VP35, pCAGGS-VP30, pCAGGS-T7, pCAGGS-Luc2 (encoding firefly luciferase), pCAGGS-L, and p4cis-vRNA-Rluc were provided by Dr. Bo Zhang (Wuhan Institute of Virology); Dr. Heinz Feldmann (National Institutes of Health, Hamilton, Montana, USA). VP40 and VP24 were generated from cDNA by PCR-based cloning techniques. VP40 was cloned into pCAGGS with an N-terminal Myc tag or C-terminal Flag tag, while VP24 was tagged with HA at the N-terminus. All mutants of NP were constructed by PCR amplification using NP as a template, and were cloned into pCAGGS with a Myc or HA tag at N-terminus. The minigenome, encoding *Renilla* luciferase, which was obtained from the plasmid p4cis-vRNA-Rluc via PCR, was generated as previously described (Muhlberger et al., 1998) and cloned into the T7 expression vector pBS, named pBS-Rluc. VP30_6A_ was generated by PCR amplification as previously described (Modrof et al., 2002). The coding region of *Sec24C* (NCBI accession no. NM_004922) was obtained from HeLa cells via RNA extraction, followed by RT-PCR, and then cloned into the pCAGGS with an HA tag at the N-terminus. The details, related to the construction process of all the plasmids, are available upon request. All the constructs were verified by DNA sequencing.

### **SDS**-**PAGE and Western blot**

Cells were harvested and lysed with 500 μL of lysis buffer (150 nmol/L NaCl, 50 nmol/L Tris-HCl (pH 7.4), 1% Triton X-100, 1 mmol/L EDTA (pH 8.0), and 0.1% sodium dodecyl sulfate (SDS)) with a protease inhibitor cocktail and then incubated on ice for 30 min. The supernatants were collected by centrifugation at 4 °C at 12,000 ×*g* for 30 min and then boiled in 5× SDS-PAGE loading buffer for 10 min. The prepared samples were resolved on 10% SDS-PAGE and then transferred onto nitrocellulose membrane (GE Healthcare). The membrane was blocked with 5% skim milk powder in phosphate-buffered saline (PBS) with 0.1% Tween 20 (PBST) for 30 min before being incubated with primary antibodies, followed by incubation with horseradish peroxidase-conjugated goat anti-rabbit or anti-mouse IgG (Thermo Fisher Scientific) for 1 h. The primary antibodies were used as follows: mouse anti-Myc (1:10,000, MBL), mouse anti-HA (1:10,000, MBL), mouse anti-Flag (1:10,000, Sigma), rabbit anti-HA (1:10,000, Sigma), rabbit anti-Flag (1:10,000, CST), rabbit anti-Myc (1:10,000, Sigma), rabbit anti-VP30 pSer29 antibody (1:1000, ABclonal), rabbit anti-Na/K ATPase (1:1000, ABclonal), and mouse anti-GAPDH (1:1000, ABclonal). The secondary antibodies, goat anti-rabbit IgG and goat anti-mouse IgG, were used at a 1:5,000 dilution.

### **Virus**-**like particle assays**

HEK293T cells were transfected with the indicated plasmids. At 36 h post-transfection, the supernatant was harvested and clarified by centrifugation at 12,000 ×*g* for 10 min and then pelleted through a 20% sucrose cushion; it was subsequently ultra centrifugated at 35,000 rpm at 4 °C for 2 h. Cell lysates were obtained as mentioned for the Western blots. The VLP pellets were resuspended in PBS at 4 °C overnight. The supernatants and cell lysates were boiled in 5× SDS-PAGE loading buffer for 10 min and then subjected to Western blot analysis.

### Protease protection assay

The VLP pellets were prepared as described above and then divided into four aliquots and treated as follows: (i) left untreated, (ii) treated with Triton X-100 to a final concentration of 1%, (iii) treated with trypsin to a final concentration of 1 μg/mL, or (iv) treated with Triton X-100 plus trypsin. The prepared samples were incubated at 37 °C for 30 min and then boiled in 5× SDS-PAGE loading buffer for 10 min and analyzed via Western blot.

### *In vivo* co-immunoprecipitation

HEK293T cells were transfected with the indicated plasmids using the calcium phosphate precipitation method. At 30 h post-transfection, cell lysates were prepared in a 500 μL lysis buffer as described for WB analysis. Then, 40 μL lysates were removed for input analysis, and the remaining cell lysates were incubated with the relevant antibodies (anti-Flag affinity gel (Sigma) or anti-Myc affinity gel (Biolegend)) overnight at 4 °C with gentle rotation. The beads were collected by centrifugation at 5,000 rpm for 2 min at 4 °C and washed three times with a lysis buffer. The collected beads and input were boiled in 1× or 5× SDS-PAGE loading buffer, respectively, for 10 min and then subjected to WB analysis.

### Immunofluorescence analysis

HeLa cells were cultured on the coverslips in 24-well plates overnight and then transfected with the indicated plasmids with Lipofectamine 2000 reagent (Invitrogen). After 26 h of transfection, the cells were harvested and fixed with ice-cold 4% (*w*/*v*) paraformaldehyde at room temperature for 20 min and then washed with PBS three times (each time for 5 min) before being incubated with 0.2% Triton X-100 for 20 min. Subsequently, the cells were blocked with 3% BSA for 30 min after being washed three times with PBS. Then, the specific primary antibodies diluted in 1% BSA were added and incubated overnight at 4 °C. After being washed by PBS, the cells were incubated with secondary antibodies diluted in 1% BSA for 1 h at room temperature. The nucleus was stained with DAPI for 5 min, and the cells were finally mounted with Prolong Diamond Antifade Mountant (Life Technology) and examined on a Leica or Zeiss confocal microscope. The primary antibodies were used as follows: rabbit anti-HA (1:1000, Sigma), mouse anti-Myc (1:1000, MBL), and mouse anti-Flag (1:1000, MBL).

### Transmission electron microscopy

HEK293T cells were transfected with the indicated plasmids. At 36 h post-transfection, the culture medium was discarded, and then the cells were fixed with a buffer (3% paraformaldehyde and 1.5% glutaraldehyde in a 0.1 mol/L sodium phosphate buffer (pH 7.4)) for 1 h at room temperature. Subsequently, the cells were harvested and subjected to gradient centrifugation at 1000 ×*g*, 3,000 ×*g*, 6,000 ×*g*, and 12,000 ×*g*. Then, the cells were fixed with 1% osmium tetroxide at 4 °C for 1 h under dark condition, followed by being incubated with 2% uranyl acetate overnight. After being dehydrated with sequential washes in 50%, 75%, 95%, and 100% ethanol, the cells were mounted with epoxy resin and then incubated overnight at 37 °C or 65 °C for 48–72 h and thinly sliced. The samples were absorbed on uncoated 200-mesh copper grids and stained with uranyl acetate and lead citrate, before being finally analyzed via transmission electron microscopy (JEOL, JEM-1400 plus).

### Live cell imaging

A previous study showed that the viral proteins of Ebola fused to larger fluorescent tags were only fully functional if the respective wild type proteins are also expressed (Takamatsu et al., 2018). Therefore, GFP-NP, GFP-NP_W699A_, or mCherry-VP40 were co-expressed with the corresponding wild type protein. For live-cell imaging, HeLa cells were grown in 20 mm dishes and transfected with the indicated plasmids. At 24 h post-transfection, live-cell time-lapse experiment images were recorded with a Zeiss LSM 800 fluorescence microscope. Pictures were taken every 120 s and processed with Zeiss Microscope Imaging Software.

### Cytosol and plasma membrane protein fractionation

HEK293T cells were transfected with the indicated plasmids. The cells were harvested at 24 h post-transfection and washed with cold PBS and then collected via low-speed centrifugation. The cytosol, organelle membrane, and plasma membrane protein fractions were isolated sequentially using the Minute^TM^ plasma membrane protein isolation and cell fractionation kit (INVENT) according to the manufacturer’s instructions. The cytosol and plasma membrane fractions were subjected to WB analysis. GAPDH and sodium potassium ATPase were used as controls for the cytosol and plasma membrane, respectively.

### Minigenome assay

HEK293T cells, cultured in a 12-well plate, were transfected with the minigenome assay system: 125 ng pCAGGS-Myc-NP, 125 ng pCAGGS-HA-VP35, 225 ng pCAGGS-Flag-VP30, 1 μg pCAGGS-L, 250 ng pCAGGS-T7 polymerase, 250 ng pBS-Rluc (minigenome, encoding *Renilla* luciferase), and 25 ng pCAGGS-Luc2 (encoding firefly luciferase as a transfection control). Total DNA levels were kept constant by transfection with pCAGGS. At 30 h post-transfection, cells were lysed in 150 μL 1× lysis buffer, and 20 μL aliquots of lysates were analyzed with the Dual-Glo Luciferase Assay System (Promega) according to the manufacturer’s instructions. A portion of the cell lysate was subjected to WB analysis as described above. Data are presented as a relative scale, with the *Renilla* luciferase activity normalized to the firefly luciferase values.

### *In vivo* RNA immunoprecipitation

HEK293T cells were grown in 6 cm dishes and transfected with 1 μg pCAGGS-Myc-NP/4 μg pCAGGS-Myc-NP_L692A_/2 μg pCAGGS-Myc-NP_P697A_/2 μg pCAGGS-Myc-NP_P698A_/4 μg pCAGGS-Myc-NP_W699A_, 1 μg pCAGGS-HAVP35, 1.8 μg pCAGGS-FlagVP30, 8 μg pCAGGS-L, 2 μg pCAGGS-T7, and 2 μg pBS-Rluc. In a separate experiment, 4 μg pCAGGS-VP40-Flag was co-expressed. pCAGGS was then transfected to keep the total DNA levels constant. After transfection for 30 h, the cells were lysed with 500 μL RNase-free NP-40 buffer (50 mmol/L Tris-HCl (pH 7.4), 50 mmol/L NaCl, 50 mmol/L NaF, 0.5% NP40) on ice for 20 min and then centrifuged at 12,000 ×*g* at 4 °C for 20 min. The supernatants were collected and divided into three parts. One portion (8%) was subjected to WB analysis to detect the expression of plasmids; another portion (12%) was resuspended in 1 mL TRIzol (Ambion, Invitrogen) to determine the total cellular minigenome RNA. The remaining cell lysates were subjected to immunoprecipitation with anti-Myc antibodies for 4 h at 4 °C. The beads were collected by centrifugation at 5,000 rpm for 2 min at 4 °C and washed 3 times with NP-40 buffer. The collected beads were finally resuspended in 1 mL NP-40 buffer and divided into two parts. One portion (6%) was collected and subjected to WB analysis to detect the amount of precipitated proteins. The remaining beads were resuspended in 1 mL TRIzol. The total cellular minigenome RNA and immunoprecipitated RNA were extracted following the manufacturer’s directions. Each sample of RNA was eluted in 20–30 μL RNase-free water. The RNA concentration was measured with a NanoDrop spectrophotometer (Promega).

### RNA quantification

The cellular and immunoprecipitated RNA of the EBOV minigenome were quantified by quantitative reverse transcription PCR (RT-qPCR). A total of 500 ng of RNA was used for the reverse transcription of genomic viral RNA (vRNA), using the kit following the manufacturer’s instructions (Fermentas, Waltham, MA, USA), followed by a qPCR assay to quantify RNA abundance. In each case, data are represented as a relative scale, with the immunoprecipitated vRNA abundance normalized to the total cellular vRNA values. The values were expressed as a percentage, where 100% was taken as abundance of immunoprecipitated Myc-NP. The specific primer for reverse transcription of the vRNA was 5′-GACAAATTGCTCGGAATCACAAAATTCC-3′ (reverse primer). The primers for the *Renilla* luciferase reporter for qPCR were as follows: 5′-GGTAGGCGTTGTAGTTGCG-3′ (forward primer); 5′-TACGAGCA CCAAGACAAGA-3′ (reverse primer).

### Statistical analysis

Data are expressed as the mean ± standard deviation (SD). The significance of the variability between different groups was determined by two-way ANOVA tests of variance using the GraphPad Prism software (version 5.0). *P* < 0.05 was considered statistically significant, and *P* > 0.05 was considered statistically non-significant.

## Electronic supplementary material

Below is the link to the electronic supplementary material.

Supplementary material 1 (PDF 4293 kb)

Supplementary material 2 (AVI 18436 kb)

Supplementary material 3 (AVI 13210 kb)

## Abbreviations

coIP: coimmunoprecipitation
COPII: coat protein complex II
EBOV: Ebola virus
F: fusion protein
GP: glycoprotein
HN: hemagglutinin-neuraminidase
HPIV1: human parainfluenza virus type 1
HPIV3: human parainfluenza virus type 3
hRSV: human respiratory syncytial virus
IBs: inclusion bodies
IF: immunofluorescence
IP-RT-qPCR: immunoprecipitation quantitative reverse transcription PCR
LLOV: Lloviu virus
M: matrix protein
MARV: Marburg virus
MeV: Measles virus
NC: nucleocapsid
NiV: Nipah virus
NNSVs: non-segmented negative strand RNA viruses
NP: nucleoprotein
NSVs: negative strand RNA viruses
P: phosphoprotein
PM: plasma membrane
PP2A-B56: protein phosphatase 2A, regulatory subunit B56 family
RNP: ribonucleoprotein
Sec24C: secretory COPII component 24C
SeV: Sendai virus
SV5: paramyxovirus simian virus 5
TEM: transmission electron microscopy
VLP: virus like particle
vRNA: genomic viral RNA
VSV: vesicular stomatitis virus
WT: wild type
