# Supplementary material for: The two-stage interaction of Ebola virus VP40 with nucleoprotein results in a switch from viral RNA synthesis to virion assembly/budding
Source: Protein Cell. 2020 Nov 3;13(2):120–40. doi: 10.1007/s13238-020-00764-0 (PMC8783937; doi:10.1007/s13238-020-00764-0)
Supplement: Supplementary file 1 — Supplementary material 1 (PDF 4293 kb) [file 13238_2020_764_MOESM1_ESM.pdf]

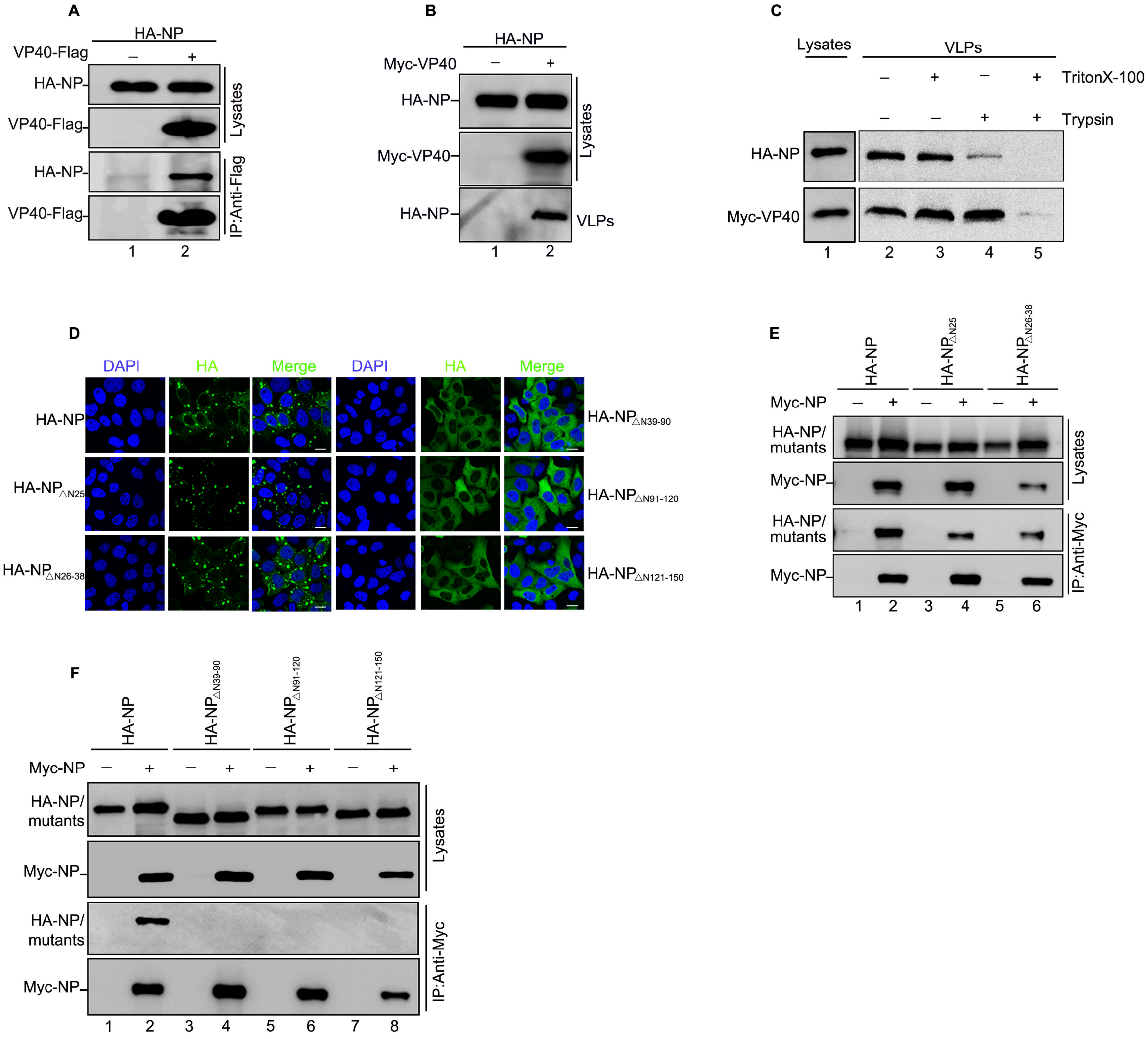
A
HA-NP
VP40-Flag
–
+
HA-NP
VP40-Flag
HA-NP
VP40-Flag
Lysates
IP:Anti-Flag
1
2
B
HA-NP
Myc-VP40
–
+
HA-NP
Myc-VP40
HA-NP
Lysates
VLPs
1
2
C
Lysates
VLPs
TritonX-100
Trypsin
HA-NP
Myc-VP40
1
2
3
4
5
D
DAPI
HA
Merge
DAPI
HA
Merge
HA-NP
HA-NP△N25
HA-NP△N26-38
HA-NP△N39-90
HA-NP△N91-120
HA-NP△N121-150
E
HA-NP
HA-NP△N25
HA-NP△N26-38
Myc-NP
HA-NP/
mutants
Myc-NP
HA-NP/
mutants
Myc-NP
Lysates
IP:Anti-Myc
1
2
3
4
5
6
F
HA-NP
HA-NP△N39-90
HA-NP△N91-120
HA-NP△N121-150
Myc-NP
HA-NP/
mutants
Myc-NP
HA-NP/
mutants
Myc-NP
Lysates
IP:Anti-Myc
1
2
3
4
5
6
7
8

Figure S1. N-terminal amino acids 39 to 150 of the NP are critical for NP oligomerization.

(A) coIP of NP with VP40. HEK293T cells were co-transfected with HA-NP and VP40-Flag. At 30 h p.t, IP was performed with anti-Flag antibodies.

(B) VLP budding assay of VP40 with NP. HEK293T cells were co-transfected with HA-NP and Myc-VP40 for 36 h. Cell lysates and VLPs were subjected to WB assay with anti-Myc, anti-HA antibodies.

(C) Protease protection assay for NP-VP40. HEK293T cells were transfected with HA-NP and Myc-VP40. The prepared VLP samples were divided into four aliquots, left untreated or treated with TritonX-100, trypsin, or Triton X-100 plus trypsin and then analyzed via WB.

(D) The subcellular localization of NP N-terminally truncated mutants. HeLa cells were transfected with HA-NP/NP mutants for 26 h, and then their distribution was analyzed by Leica microscopy. Scale bars=10μm.

(E-F) HEK293T cells were co-transfected with Myc-NP and HA-NP/NP N-terminal mutants for 30 h. Cell lysates were subjected to IP with anti-Myc antibodies. The coIP of NP with $NP_{\triangle N25}$ or $NP_{\triangle N26-38}$ (E); the coIP of NP with $NP_{\triangle N39-90}$, $NP_{\triangle N91-120}$, or $NP_{\triangle N121-150}$ (F).

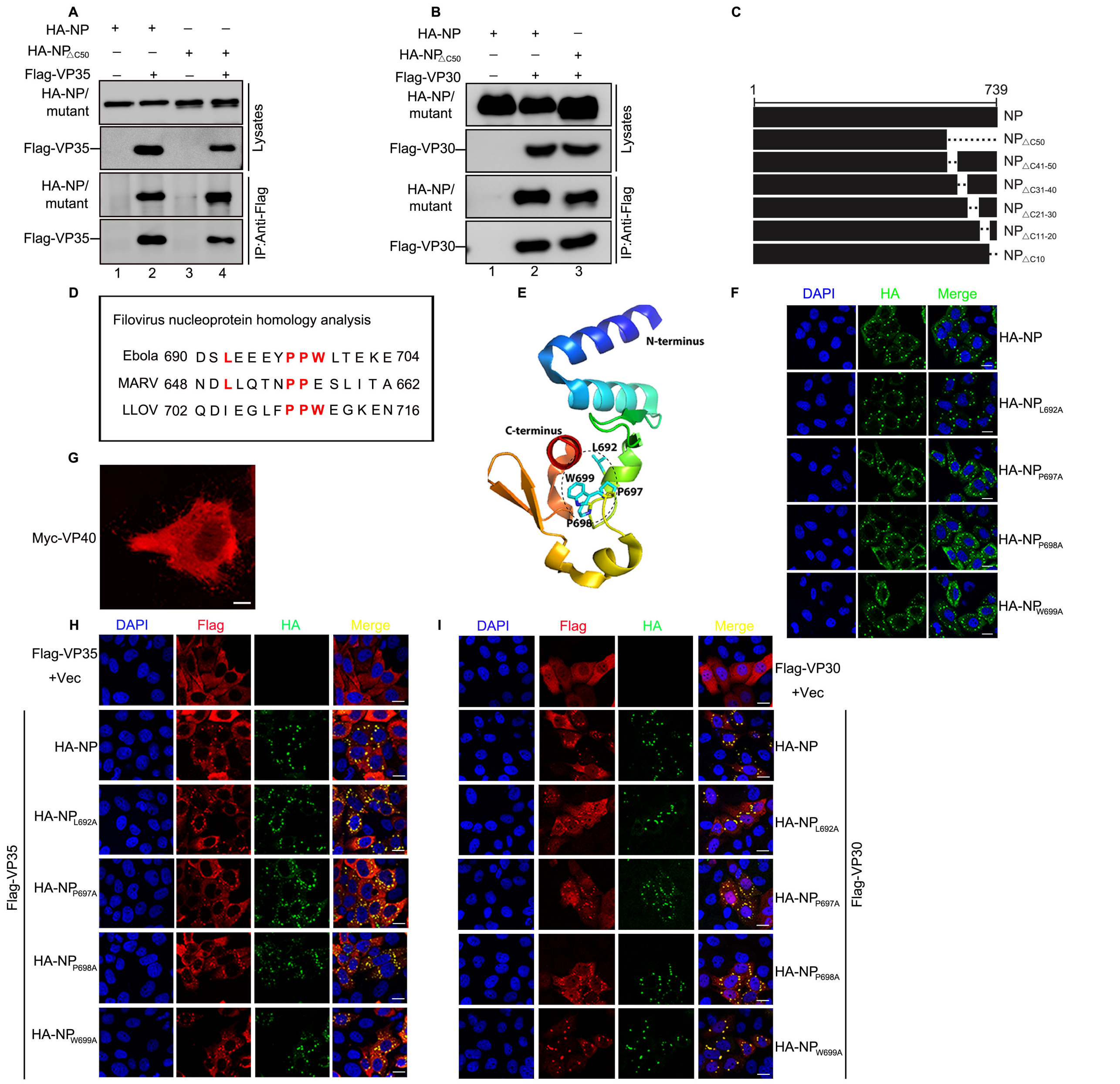

A
HA-NP
HA-NP△C50
Flag-VP35
HA-NP/ mutant
Flag-VP35
HA-NP/ mutant
Flag-VP35
Lysates
IP:Anti-Flag
1 2 3 4
B
HA-NP
HA-NP△C50
Flag-VP30
HA-NP/ mutant
Flag-VP30
HA-NP/ mutant
Flag-VP30
Lysates
IP:Anti-Flag
1 2 3
C
1
739
NP
NP△C50
NP△C41-50
NP△C31-40
NP△C21-30
NP△C11-20
NP△C10
D
Filovirus nucleoprotein homology analysis
Ebola 690 D S L E E E Y P P W L T E K E 704
MARV 648 N D L L Q T N P P E S L I T A 662
LLOV 702 Q D I E G L F P P W E G K E N 716
E
N-terminus
C-terminus
L692
W699
P697
P698
F
DAPI
HA
Merge
HA-NP
HA-NPL692A
HA-NPP697A
HA-NPP698A
HA-NPW699A
G
Myc-VP40
H
DAPI
Flag
HA
Merge
Flag-VP35 +Vec
HA-NP
HA-NPL692A
HA-NPP697A
HA-NPP698A
HA-NPW699A
Flag-VP35
I
DAPI
Flag
HA
Merge
Flag-VP30 +Vec
HA-NP
HA-NPL692A
HA-NPP697A
HA-NPP698A
HA-NPW699A
Flag-VP30

Figure S2. The characteristics of $NP_{\triangle C50}$, NP_{L692A}, NP_{P697A}, NP_{P698A} and NP_{W699A}.

(A) coIP of $NP_{\triangle C50}$ with VP35. HEK293T cells were co-transfected with HA-NP/ $NP_{\triangle C50}$ and Flag-VP35 for 30 h. Cell lysates were subjected to IP with anti-Flag antibodies.

(B) coIP of $NP_{\triangle C50}$ with VP30. HEK293T cells were co-transfected with HA-NP/ $NP_{\triangle C50}$ and Flag-VP30 for 30 h. Cell lysates were subjected to IP with anti-Flag antibodies.

(C) Schematic diagrams of wild type NP and its C-terminally truncated mutants.

(D) The homology analysis of NP C-terminus among the filoviruses.

(E) The structure analysis of NP C-terminus. The side chains of L692, P697, P698 and W699 are shown by a stick model, and the elliptical dotted line indicates the hydrophobic core formed by their side chains (PDB:4QB0).

(F) HeLa cells were transfected with HA-NP/ NP C-terminal point mutants for 26 h, and analyzed for the distribution of NP/NP C-terminal mutants by Leica microscopy. Scale bars=10μm.

(G) HeLa cells were transfected with Myc-VP40 for 26 h and analyzed for the distribution of VP40 by superresolution microscopy (Zeiss LSM 800). Scale bars= 5μm.

(H) HeLa cells were transfected with Flag-VP35, individually or co-transfected with HA-NP/NP C-terminal point mutants for 26 h. The co-localization of VP35 with NP/NP C-terminal mutants was analyzed on Leica microscopy. Scale bars=10μm.

(I) HeLa cells were transfected with Flag-VP30, individually or co-transfected with HA-NP/NP C-terminal point mutants for 26 h. The co-localization of VP30 with NP/NP

C-terminal mutants was analyzed on Leica microscopy. Scale bars=10μm.

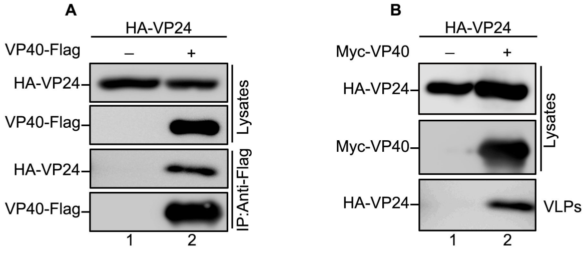

A
HA-VP24
VP40-Flag
–
+
HA-VP24
VP40-Flag
HA-VP24
VP40-Flag
Lysates
IP:Anti-Flag
1
2
B
HA-VP24
Myc-VP40
–
+
HA-VP24
Myc-VP40
HA-VP24
Lysates
VLPs
1
2

Figure S3. VP24 can interact with VP40 and be incorporated into VP40–VLP.

(A) coIP of VP24 with VP40. HEK293T cells were co-transfected with HA-VP24 and VP40-Flag. At 30 h p.t, IP was performed with anti-Flag antibodies.

(B) VLP budding assay of VP40 with VP24. HEK293T cells were co-transfected with HA-VP24 and Myc-VP40 for 36 h. Cell lysates and VLPs were subjected to WB assay with anti-Myc, anti-HA antibodies.

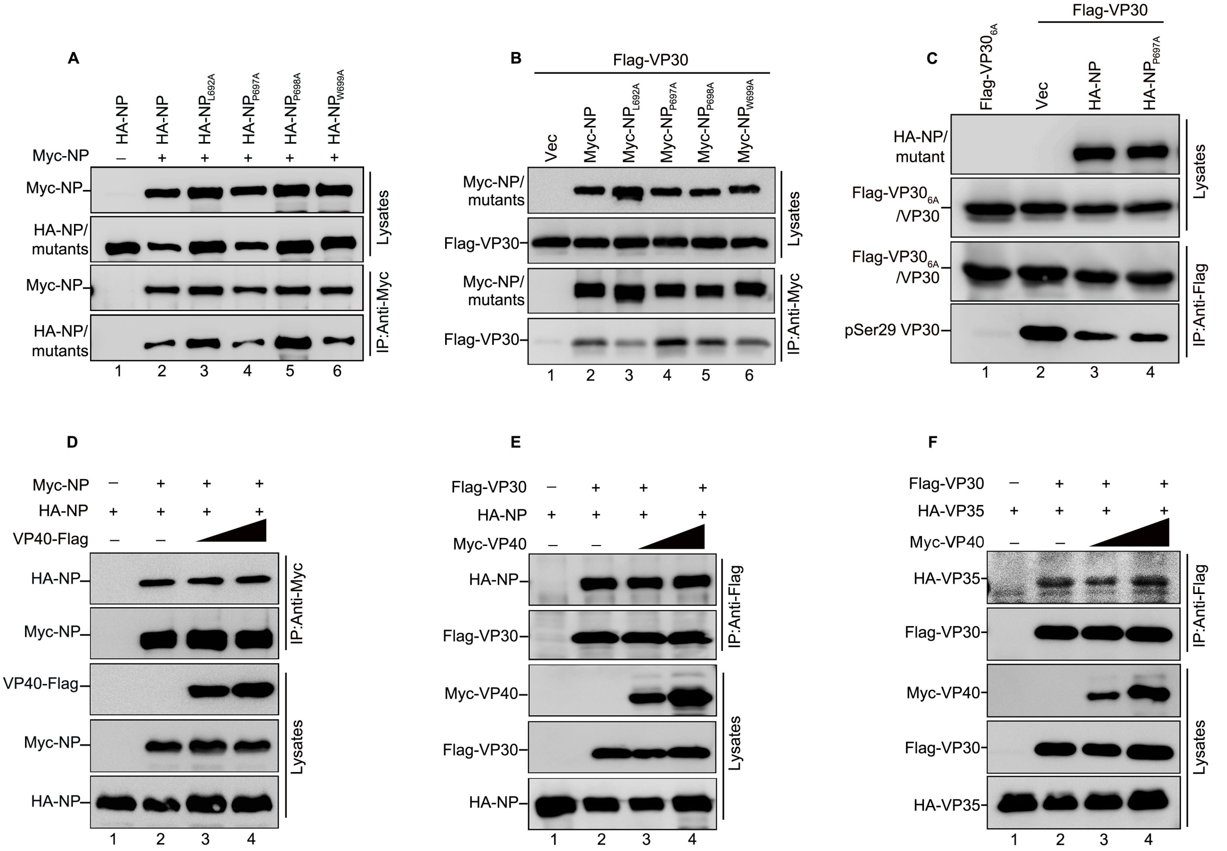

A
HA-NP
HA-NP
HA-NP$_{L692A}$
HA-NP$_{P697A}$
HA-NP$_{P698A}$
HA-NP$_{W699A}$
Myc-NP – + + + + +
Myc-NP
HA-NP/mutants
Myc-NP
HA-NP/mutants
Lysates
IP:Anti-Myc
1 2 3 4 5 6
B
Flag-VP30
Vec
Myc-NP
Myc-NP$_{L692A}$
Myc-NP$_{P697A}$
Myc-NP$_{P698A}$
Myc-NP$_{W699A}$
Myc-NP/mutants
Flag-VP30
Myc-NP/mutants
Flag-VP30
Lysates
IP:Anti-Myc
1 2 3 4 5 6
C
Flag-VP30$_{6A}$
Flag-VP30
Vec
HA-NP
HA-NP$_{P697A}$
HA-NP/mutant
Flag-VP30$_{6A}$ /VP30
Flag-VP30$_{6A}$ /VP30
pSer29 VP30
Lysates
IP:Anti-Flag
1 2 3 4
D
Myc-NP – + + +
HA-NP + + + +
VP40-Flag – –
HA-NP
Myc-NP
VP40-Flag
Myc-NP
HA-NP
IP:Anti-Myc
Lysates
1 2 3 4
E
Flag-VP30 – + + +
HA-NP + + + +
Myc-VP40 – –
HA-NP
Flag-VP30
Myc-VP40
Flag-VP30
HA-NP
IP:Anti-Flag
Lysates
1 2 3 4
F
Flag-VP30 – + + +
HA-VP35 + + + +
Myc-VP40 – –
HA-VP35
Flag-VP30
Myc-VP40
Flag-VP30
HA-VP35
IP:Anti-Flag
Lysates
1 2 3 4

Figure S4. The functions of NP$_{L692A}$, NP$_{P697A}$, NP$_{P698A}$ and NP$_{W699A}$ and the effects of VP40 on nucleocapsid formation.

(A) coIP assay of the NP with NP$_{L692A}$, NP$_{P697A}$, NP$_{P698A}$ or NP$_{W699A}$. HEK293T cells were co-transfected with HA-NP/NP C-terminal mutants and Myc-NP for 30 h. Cell lysates were subjected to IP with anti-Myc antibodies and analyzed via WB using anti-HA, anti-Myc antibodies.

(B) coIP assay of the Flag-VP30 with Myc-NP/NP$_{L692A}$, NP$_{P697A}$, NP$_{P698A}$ or NP$_{W699A}$. HEK293T cells were transfected with the indicated plasmids. Cell lysates were subjected to IP with anti-Myc antibodies and analyzed via WB using anti-Flag, anti-Myc antibodies.

(C) The effect of NP$_{P697A}$ on the levels of VP30 phosphorylation. HEK293T cells were co-transfected with HA-NP/NP$_{P697A}$ and Flag-VP30 for 30 h. Flag-VP30$_{6A}$ (six serines at N-terminus were mutated to alanines) was transfected as a negative control. IP was performed with anti-Flag antibodies. The levels of VP30 phosphorylation were detected using VP30 pSer29 antibodies.

(D) The effect of VP40 on NP oligomerization. HEK293T cells were co-transfected with Myc-NP, HA-NP, and increasing amounts of VP40-Flag for 30 h. Cell lysates were subjected to IP with anti-Myc antibodies and analyzed via WB.

(E-F) The effect of VP40 on the interactions of NP-VP30 or VP30-VP35. (E) HEK293T cells were co-transfected with Flag-VP30, HA-NP, and increasing amounts of Myc-VP40. IP was performed with anti-Flag; (F) Flag-VP30, HA-VP35, and increasing amounts of Myc-VP40 were co-transfected into HEK293T cells. IP was performed with

anti-Flag.

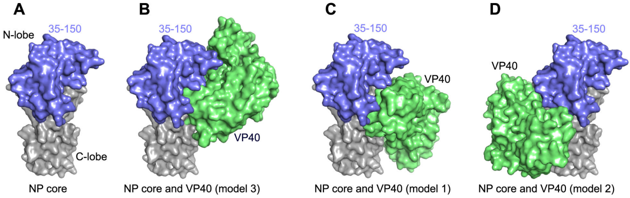
A
35-150
N-lobe
C-lobe
NP core
B
35-150
VP40
NP core and VP40 (model 3)
C
35-150
VP40
NP core and VP40 (model 1)
D
VP40
35-150
NP core and VP40 (model 2)

Figure S5. The structural analysis of the NP N-terminus and VP40 interacting regions.

(A) The structural analysis was performed using the HDOCK server, based on the NP core structure (PDB: 4Z9P; residues from 35-350; as the receptor molecular), and the VP40 structure (PDB: 1ES6; residues from 31-326; as the ligand molecular). Residues 35-150 of NP that form one face of the N-lobe was specified as the binding site. Blue indicates the N-lobe of NP; gray indicates the C-lobe of NP; green indicates VP40.

(B-D) The top three predicted results were analyzed. (B) VP40 binds to N-lobe of NP, and this model with the third highest docking score, but exhibited the largest interacting area of the three; (C-D) the top two models have interacting region with the C-lobe.
